# Surface strain at the cervical area and fracture strength of flared root canals reinforced using a zirconia tube and glass-fiber post

**DOI:** 10.1016/j.jds.2023.12.012

**Published:** 2024-01-08

**Authors:** Daiki Kondo, Wataru Komada, Shinya Oishi, Kenji Fueki

**Affiliations:** Division of Masticatory Function and Health Science, Graduate School of Medical and Dental Sciences, Tokyo Medical and Dental University, Tokyo, Japan

**Keywords:** Zirconium oxide, Fracture strength, Endodontically treated teeth

## Abstract

**Background/purpose:**

Recently, an effective core build-up system for teeth with flared root canals is needed. This research aimed to evaluate the effect of foundation restorations using a composite resin core with a fiber post reinforced with a zirconia tube for the surface strain at the cervical area and the fracture load of teeth with flared root canals.

**Materials and methods:**

Bovine teeth were shaped to mimic human premolars with flared root canals and restored using three types of composite resin foundation restorations with each materials described below: a fiber post (FC), a zirconia tube (ZC), a fiber post and zirconia tube (ZFC). Each specimen was restored with a zirconia crown. The surface strains of the specimens at the cervical area and fracture loads were analyzed using a one-way analysis of variance (ANOVA), followed by Tukey's honest significant difference test.

**Results:**

The surface strains of Groups ZFC and ZC were significantly lower than that of Group FC in the buccal root. The fracture strengths of Groups ZFC and ZC were significantly higher than that of Group FC. The strength of Group ZFC was significantly higher than that of Group ZC.

**Conclusion:**

The use of a composite resin core with a zirconia tube for the simulated premolar with flared root canals reduced surface strain at the cervical area and provided higher fracture strength compared to using a composite resin core with a fiber post. And the zirconia tubes provided even higher fracture strength when used with a fiber post.

## Introduction

Post-and-cores are frequently used to restore endodontically treated teeth that have lost a significant amount of coronal tooth structure. Cast post-and-cores are widely used owing to their superior mechanical properties.^1^^,^^2^ However, root fractures are frequently observed in seriously damaged teeth restored using cast post-and-cores because of the difference between the elastic moduli of the root dentin and metal post.^3^, ^4^, ^5^, ^6^ Composite resins have been as a substitute to cast post-and-cores in foundation restorations following significant developments in dental materials, such as improved adhesion.^7^ Glass-fiber posts are applied to strengthen the tooth structure in cases where seriously damaged teeth are restored using composite resin.^8^, ^9^, ^10^ Foundation restorations using a composite resin and prefabricated glass-fiber posts reduce the possibility of vertical root fractures as their elastic moduli are comparable to that of dentin.^6^^,^^11^, ^12^, ^13^

The concentration of stress is observed in the cervical area of teeth that have been restored using composite resin core with prefabricated glass-fiber posts^14^, ^15^, ^16^ which can result in horizontal fractures and debonding of the post-and-core.^16^, ^17^, ^18^ The concentration of stress in the cervical area also causes delamination of the post-and-core and the root dentin, which in turn leads to their fracture.^17^^,^^19^ Guzy et al. reported that greater stresses are observed near the root surface than at the center of the teeth when occlusal forces are applied to endodontically treated teeth.^20^ Therefore, reinforcement of the cervical area is considered an effective strategy to improve the fracture strength of endodontically treated teeth. Consequently, zirconia tubes were developed as a new material in this research to reinforce the cervical surface area of endodontically treated teeth. Zirconia has high fracture strength, biocompatibility, and high adhesion to composite resins and is used for crown restoration.^21^^,^^22^

The stress concentrated in the cervical surface area of a model of a mandibular molar with large pulp chamber made of a composite resin was reduced applying a prefabricated zirconia tube and composite resin post-and-core in a previous study.^23^ However, it is unclear whether a similar effect can be observed in teeth other than the mandibular first molars. It is also unclear how the use of zirconia tubes in combination with composite resin post-and-cores affects the fracture strength.

Anterior teeth and premolars which have endodontically treated several times often have flared root canals, and in that case the teeth have thin dentin wall and are considered to be at high risk for root fracture.^24^ When such teeth are restored with composite resin core, it has been reported that a fiber post positioned only at the central area of the post space does not significantly improve the fracture strength.^24^ And an effective metal-free restoration to improve the strength of flared root canals has not been established. The reason why the fracture strength is not significantly improved by reinforcing only the center of the post space is thought to be that stresses concentrate near the root surface than at the center of the teeth when occlusal forces are applied to endodontically treated teeth.^20^

Therefore, this research aimed to evaluate the effect of foundation restorations employing a composite resin core and a fiber post reinforced with a zirconia tube for the strain at the cervical surface area and the fracture load of endodontically treated teeth with flared root canals. The null hypothesis was that applying a zirconia tube in endodontically treated teeth with flared root canals restored using composite resin core with a fiber post would not reduce the surface strain at the cervical area and provide higher fracture strength.

## Materials and methods

24 bovine mandibular incisors which have no cracks or fractures were used in this research. The reason for using bovine teeth was to standardize the root morphology. The mechanical properties and bond strength of bovine teeth to composite resin are similar to those of human teeth.^25^^,^^26^ Bovine teeth were stored at −15 °C and defrosted at 25 °C before the experiment. The periodontal tissue was removed, and the root length was adjusted to 13 mm from the apex with a low-speed diamond saw (Isomet, Buehler Ltd., Lake Bluff, IL). All roots were uniformly shaped on a lathe (KS-310; Toyo Associates Ltd., Tokyo, Japan) to mimic the roots of human mandibular premolars. The root canals were enlarged to No. 120 using a K-file (Files K, GC Co., Tokyo, Japan). The root canals were rinsed with 2.5–3.5 % hydrogen peroxide solution and 6 % sodium hypochlorite solution, dried using a paper point, and filled with a root canal obturation material (Gutta Percha Points, GC Co.) and a zinc oxide non-eugenol root canal sealer (Canals N, Showa Yakuhin Kako Co., Ltd., Tokyo, Japan). The specimens were stored in the dark in water for 24 h. Subsequently, all root canals were prepared to a post length of 8 mm with a diamond point (H250 033; Horico, Berlin, Germany). The thickness of the dentin wall in the cervical area was adjusted to 0.8 mm using a carborundum point (HP No. 44; Shofu Inc., Kyoto, Japan). The morphology of the post space was determined according to a previous study.^27^

**Figure 1:**
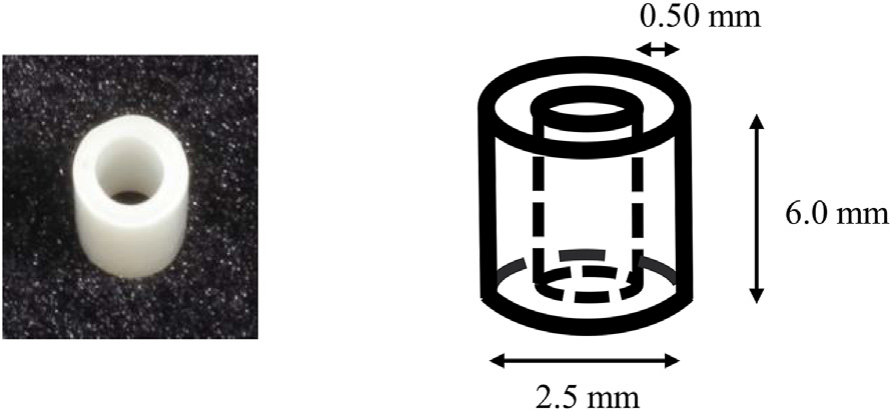
The shape of a prefabricated zirconia tube.

Zirconia tubes (diameter, 2.5 mm; height, 6 mm; and thickness, 0.5 mm) prepared from zirconia discs (Katana HTML, Kuraray Noritake Dental Inc., Tokyo, Japan) were used in this study (Fig. 1).

**Figure 2:**
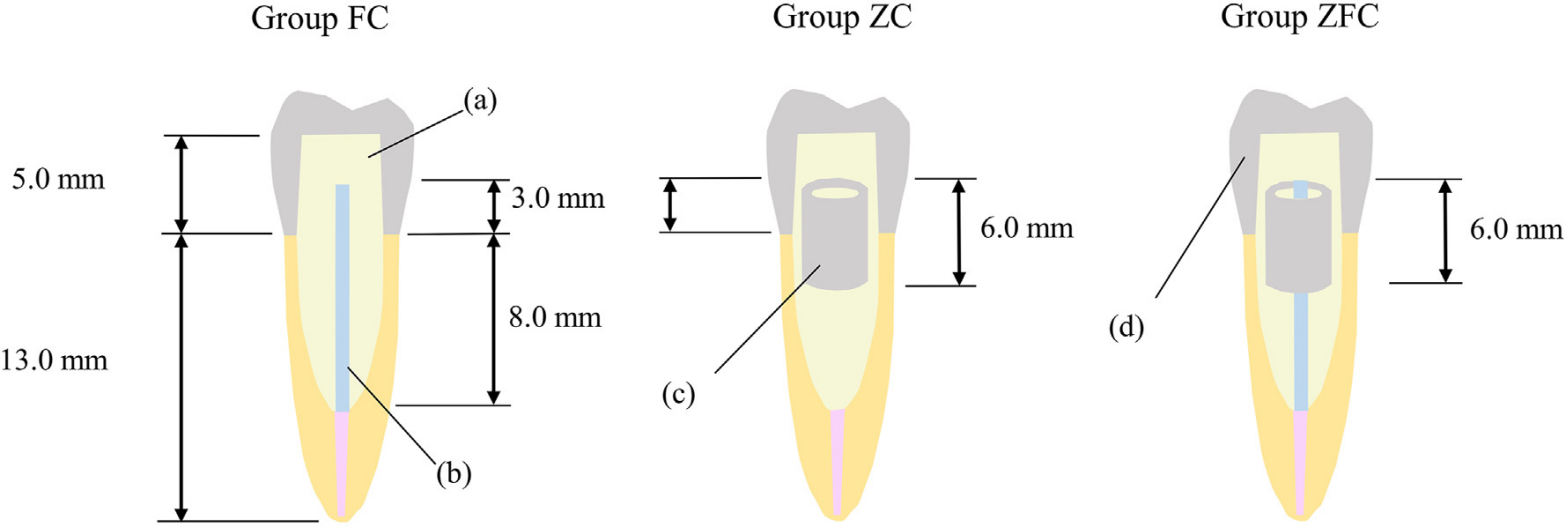
The experimental groups. FC: composite resin core with prefabricated fiber post, ZC: composite resin core with a prefabricated zirconia tube, ZFC: composite resin core with a prefabricated zirconia tube and prefabricated fiber post. (a) Composite resin, (b) Prefabricated glass-fiber post, (c) Prefabricated zirconia tube, (d) and Monolithic zirconia crown.

Three different core build-up systems were tested in this study: a composite resin core and a glass-fiber post (FC), a composite resin core and a zirconia tube (ZC), and a composite resin core along with a zirconia tube and a glass-fiber post (ZFC). Eight specimens were allocated to each group for each test as described in previous studies.^2^^,^^23^ In the previous study, the mean and standard deviation measured in the pilot study were used to calculate effect sizes and determine sample sizes.^23^ Impressions of the post space of the specimens were made using a silicone impression material (Examixfine regular type and Exafine putty type, GC Co.), and a dental plaster cast was fabricated from the dental stone (New Fujirock, GC Co.). A core pattern was waxed up on the plaster cast, and an impression was made using an additional reactive silicone impression material (MEMOSIL2, Heraeus Kulzer, Hanau, Germany) to create a mold of the core pattern. The abutment height was adjusted to 5 mm. The surfaces of the experimental roots were cleaned with distilled water and air-dried. A bonding agent (Clearfil Universal Bond Quick ER, Kuraray Noritake Dental Inc.) was applied for 10 s and spread over the entire post space using a low-pressure air blow. The excess bonding agent was removed using paper points and air blow. The surfaces of the post spaces were cured for 20 s using a light-curing unit (Blueshot, Shofu Inc.), which emitted light at an intensity of 650 mW/cm^2^ (Fig. 2).

In Group FC, the prefabricated glass-fiber posts (Clearfil AD Fiber Post Ⅱ #3, Kuraray Noritake Dental Inc.) were sectioned at a point 13 mm from the apex side, applied 40 % phosphoric acid gel (K-etchant GEL, Kuraray Noritake Dental Inc.) for 5 s, and rinsed with distilled water. The posts were air-dried subsequently and coated with a ceramic primer (Clearfil Ceramic Primer Plus, Kuraray Noritake Dental Inc.). An automix composite resin (Clearfil DC Core Automix One, Kuraray Noritake Dental Inc.) was infused into the post spaces of the roots. The glass-fiber posts were inserted into the post spaces subsequently and light-cured from the occlusal direction for 20 s. The composite resin was injected into the mold, and the specimens were pressed against the mold and light-cured for 20 s each in the occlusal, buccolingual, and proximal directions. The specimens were then stored for 24 h at 37 °C with 100 % humidity.

In Group ZC, the surfaces of the zirconia tubes were sandblasted at 0.2 MPa^28^ maintaining the distance of 10 mm for 10 s using 70 μm alumina (HIALUMINAS, Shofu Inc.). The zirconia tubes were coated with a ceramic primer containing MDP (Clearfil Ceramic Primer Plus, Kuraray Noritake Dental Inc.) and air-dried. The application of MDP-containing ceramic primers improves the strength of the bond between composite resin and zirconia.^29^ Composite resin was infused into the post spaces and zirconia tubes, and the zirconia tube was inserted into the post spaces 3 mm below the margin and light-cured from the occlusal direction for 20 s. The abutment was built and the specimens were stored as described above.

In Group ZFC, surface treatment of the zirconia tube and glass-fiber post was performed as described above. The glass-fiber posts were inserted into the zirconia tubes after injecting the composite resin into the post spaces and zirconia tubes. The glass-fiber posts and zirconia tubes were inserted into the post spaces 3 mm above the margin and light-cured from the occlusal direction for 20 s. The abutment was built and the specimens were stored as described above.

The core was scanned with an extraoral scanner (AutoScan DS-EX Pro, Shining 3D Tech. Co., Ltd., Hangzhou, China). A master crown was cemented to an abutment tooth with the same morphology as that of the experimental specimens. The master crown was scanned using the extraoral scanner, and crowns were designed for each specimen using the double-scanning method. The minimum crown thickness was programmed to be 0.5 mm. Monolithic zirconia crowns were prepared from zirconia disks (Katana HTML, Kuraray Noritake Dental Inc.) with a 5-axis milling machine (MD 500, Canon Electronics Inc., Tokyo, Japan) and introduced into a speed-sintering furnace (inFire HTC, Dentsply Sirona, York, PA, USA).

The inner surfaces of the crowns were sandblasted at 0.2 MPa maintaining the distance of 10 mm for 10 s using 70 μm alumina, coated with ceramic primer containing MDP (Clearfil Ceramic Primer Plus, Kuraray Noritake Dental Inc.), and air-dried. A tooth primer (Panavia V5 tooth primer, Kuraray Noritake Dental Inc.) was applied to the abutments, and the abutments were air-dried. The crowns were cemented to the specimens using dual-cure resin cement subsequently (Panavia V5, Kuraray Noritake Dental Inc.).

**Figure 3:**
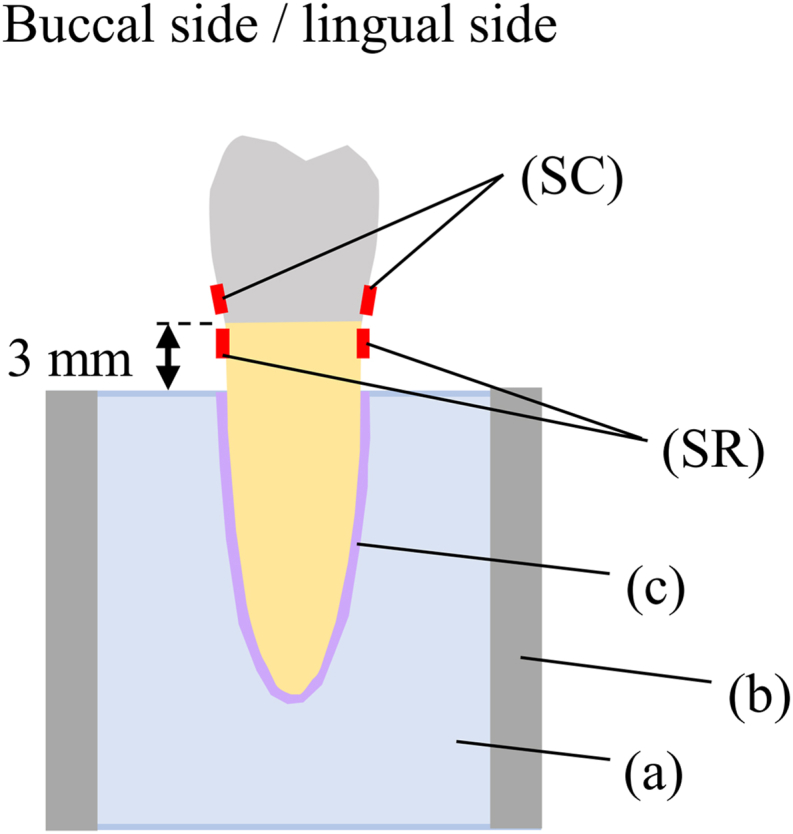
Schematic representation of the embedded experimental specimens. (a) Acrylic resin, (b) Aluminum ring, (c) Vinyl polysiloxane impression material, (SC) Strain gauge attached to crown, and (SR) Strain gauge attached to root.

Four strain gauges (KFRB-02N-120-C1-16 N30C2; Kyowa Electronic Instruments Co., Ltd., Tokyo, Japan) were adhered to the crowns and roots immediately above and below the margin on the buccal and lingual sides to measure the surface strain in the cervical area (Fig. 3). It is because when single-root teeth were subjected to a load applied at 45°angle to the long axis, it was reported that the greatest compressive and tensile stresses occur at the lingual or buccal surface of the cervical area.^30^ The adhered surfaces were sandblasted at 0.2 MPa using 70 μm alumina maintaining the distance of 10 mm for 5 s. A strain gauge cement (CC-33A, Kyowa Electronic Instruments Co., Ltd.) was applied to the bonding surfaces, and the strain gauges were held for 1 min under finger pressure. The samples were stored for 24 h at room temperature. The roots of the samples were embedded into acrylic resin (Palapress vario; Heraeus Kulzer.) up to 3 mm below the margin. The roots were surrounded with a vinyl polysiloxane material (Correct Quick, Pentron Co., Wallingford, CT, USA) which has a thickness of 0.25 mm to simulate the periodontal ligament. Loads were applied at 45° to the long axis with a universal testing machine (Autograph AGS-H, Shimadzu Co., Kyoto, Japan) at a crosshead speed of 1.0 mm/min up to 50 N using a ball end with a diameter of 2 mm. The loading point was the inner slope of the buccal cusp. The strain in the buccolingual cervical area was also measured. The data obtained were corrected using the gauge rate. It was reported that the mean human masticatory force is approximately 50 N.^31^ The magnitude of the load was set within the range of the loads applied to the premolars during masticatory function.^32^

**Figure 4:**
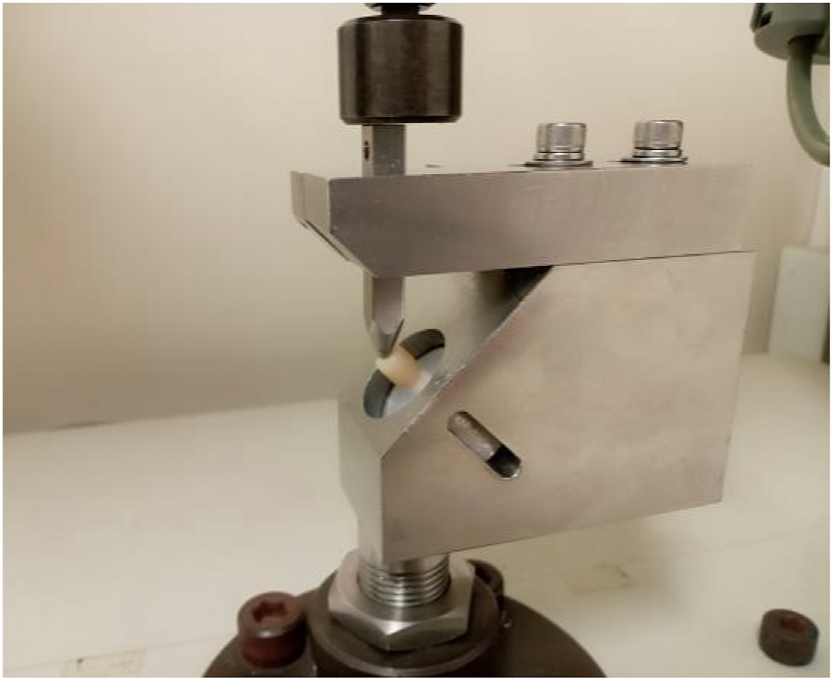
Experimental set up for the fracture test.

The fracture loads of the specimens were measured as described below. Loads were applied to the inner slope of the buccal cusp at 45° to the long axis of the tooth using the universal testing machine (Autograph AGS-H, Shimadzu Co.) at a crosshead speed of 1.0 mm/min to measure the maximum load at failure (Fig. 4). The load with 45° direction simulated the oblique load applied to the teeth during masticatory function.^32^ The reason for applying the oblique load was that oblique load provides higher strain at the cervical surface area than axial load.^32^

To evaluate the surface strain, the effect of the type of foundation restoration on the values at each measurement site was assessed using a one-way analysis of variance (ANOVA), and post hoc multiple comparisons were made using Tukey's honest significant difference (HSD) test. To evaluate the fracture strength, the effect of the type of foundation restoration on the value was assessed using a one-way ANOVA, and post-hoc multiple comparisons were made using Tukey's HSD test. Statistical significance was set at *P* < 0.05. All statistical analyses were performed using Statistical Package for the Social Sciences (SPSS version 22.0; IBM Corp., Armonk, NY, USA).

## Results

**Figure 5:**
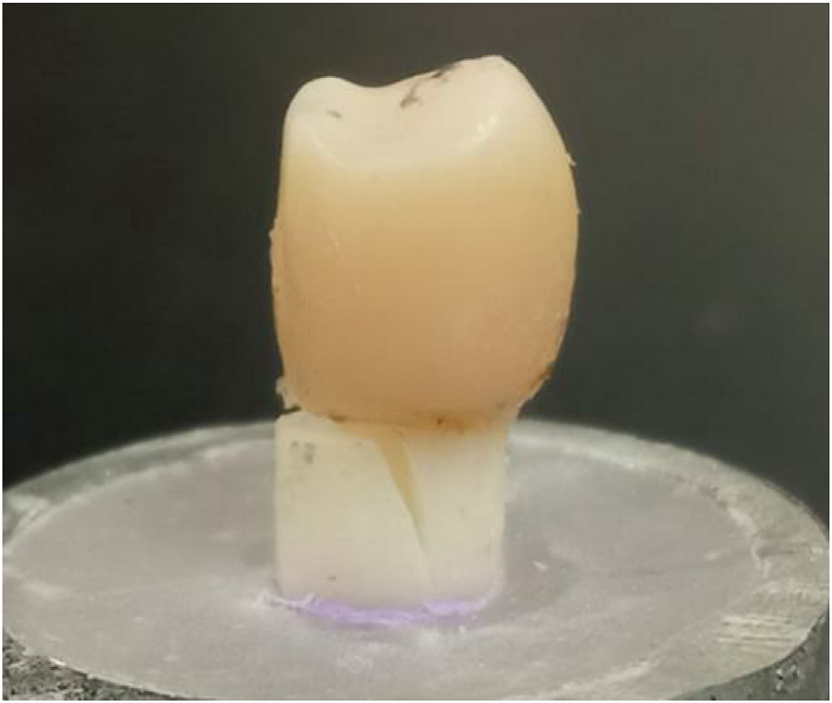
A photograph of the fractured specimen.

Table 1 shows the mean values and standard distributions of the surface strains. One-way ANOVA revealed a significant difference in the surface strains on the buccal aspect of the root (F = 17.8, *P* < 0.001). Tukey's HSD test revealed that the surface strains on the buccal aspect of the root in Groups ZFC and ZC were significantly lower than that in Group FC (*P* < 0.001). One-way ANOVA revealed significant intergroup differences in fracture strength (F = 14.9, *P* < 0.001). Tukey's HSD test revealed that the mean (standard deviation) fracture strengths of Groups ZFC [1286 (195) N] and ZC [1013(219) N] were significantly higher than that of Group FC [777(135) N] (*P* < 0.05). In addition, it was observed that the strength of Group ZFC was significantly higher than that of Group ZC (*P* = 0.021). However, irreparable vertical root fractures extending below the alveolar bone margin were observed in all specimens (Fig. 5).

**Table 1:** Mean (standard deviation) of the surface strain (με) in the experimental groups (n = 8).

Group	Crown		Root	
	Buccal	Lingual	Buccal	Lingual
FC	-28.26(18.42)	4.71(9.92)	-701.36(164.03)^a^	312.81(172.78)
ZC	-18.80(16.10)	6.29(10.74)	-361.59(125.05)^b^	210.55(168.54)
ZFC	-16.34(12.99)	3.53(8.42)	-364.21(95.30)^b^	253.39(151.78)

Different superscript letters indicate significant difference on each loading point in each strain gauge (*P* < 0.05).

Positive and negative values of the measured data indicate tensile and compressive strain, respectively.

FC: composite resin core with prefabricated fiver post, ZC: composite resin core with a prefabricated zirconia tube, ZFC: composite resin core with a prefabricated zirconia tube and prefabricated fiver post.

## Discussion

This research evaluated the effects of three foundation restorations on the surface strain and fracture strength of the cervical area of teeth which have flared root canals with no ferrules.

The null hypothesis that applying a zirconia tube in endodontically treated teeth which have flared root canals restored using composite resin core with a fiber post would not reduce the strain at the cervical surface area and provide higher fracture strength.

A large compressive strain tended to occur on the root surface of the buccal aspect near the load side when the surface strain in the cervical area was measured. Significant differences were observed between the surface strains on the root surface of the buccal aspect in Groups ZFC and FC and between Groups ZC and FC, and the strains in Groups ZFC and ZC were smaller than that in Group FC (*P* < 0.001). This result may be attributed to the root surface of the buccal aspect, where stress is most concentrated, being reinforced from the inside with a zirconia tube which has a high elastic modulus to restrain the deformation of the cervical area. No significant differences were observed among the three groups in terms of the surface strains on the buccal aspect of the crown, lingual aspect of the crown, or lingual aspect of the root. Guzy et al. reported that the stress near the root surface is greater than that at the center in endodontically treated teeth.^20^ The reason for this result may be that the zirconia crowns, which have a higher elastic modulus, were located outside the post-and-core. Thus, the zirconia crowns influenced the cervical surface strain more strongly than the core-build-up system. The surface strains on the lingual side of the root in Groups ZC and ZFC tended to be less than that in Group FC; however, this effect was not significant as the surface strain values were smaller than those on the buccal side, which was closer to the loading side.

The results of the fracture test revealed that the fracture loads exhibited by Groups ZFC and ZC were found to be significantly higher in comparison to that observed in Group FC. Stress concentrates in the cervical area and causes delamination of the root dentin and post-and-core when occlusal force is applied to an endodontically treated tooth restored using a composite resin core; this is considered the starting point for fractures of root dentin and foundation restorations.^17^ The fracture loads exhibited by Groups ZFC and ZC were found to be higher in comparison to that observed in Group FC as the zirconia tubes reinforced the thin dentin wall of the root canal, reduced the deformation of the cervical area, thereby increasing the resistance to root dentin and post-and-core fractures occurring from the cervical area.

The fracture load exhibited by Group ZFC was found to be significantly higher in comparison to that observed in Group ZC. The high elastic modulus of zirconia may lead to stress being concentrated at the lower end of the zirconia tubes, similar to the stress concentration at the tips of metal posts.^15^ Barcelos et al. reported that fiber posts can channel the stress concentrated in the crown to the root,^13^ and Michael et al. reported that fiber posts can relieve stress on the adhesive interface between the root dentin and foundation restoration and prevent delamination.^19^ The fracture load of Group ZFC being higher than that of Group ZC was thought to be due to the fiber post distributing the stress concentrated at the lower end of the zirconia tube downward toward the root, relieving the stress at the adhesive interface between the foundation restoration and root dentin. Junge et al. suggested that immoderate surface strain at the margins of crowns can cause the fracture of the adhesive between the root dentin and foundation restoration.^33^ The loss of adhesion between the root dentin and foundation restoration is considered the starting point for progressive fracture of the root dentin and post-and-core.^17^^,^^19^ In addition, the fracture of the root dentin and post-and-core in the cervical area can cause root fracture and debonding of the foundation restoration.^10^ The surface strain and fracture load measurements of the cervical area obtained in this study indicate that reinforcing the cervical surface of teeth which have flared root canal with zirconia tubes reduced the surface strain on the buccal aspect of the root in the cervical area and improved fracture strength. In addition, the use of a composite resin core with a zirconia tube and a fiber post as a foundation restoration resulted in an improvement in fracture strength compared with using a composite resin core and a zirconia tube. These results suggest that the zirconia tubes with fiber posts can be used in a metal-free core build-up system that prevents root fracture or debonding of the foundation restoration in the teeth which have flared root canals.

The zirconia tube used in this experiment was fabricated using 3Y-TZP zirconia, which is the same material used for fabricating crowns. As the minimum thickness required to fabricate a crown using 3Y-TZP zirconia is 0.5 mm,^34^ the thickness of the zirconia tube was set as 0.5 mm. The height of the zirconia tube was set as 6 mm to allow for the same length of insertion into the core and root, according to the 3 mm insertion of the fiber post into the core in a previous study.^24^^,^^27^

This study has some limitations. First, the magnitudes of the strain and fracture strength were measured by applying static loads to the restored teeth in this study. However, the clinical environment differs from the experimental conditions used in this study. Second, the oral environment is moist due to the presence of saliva, and the temperature is not constant. Third, the load applied to the teeth by mastication is much smaller than that applied in the fracture resistance test in this study; however, it is repeated. Repeated loading during mastication has been reported to cause delamination and microcracks at the adhesive interface between the foundation restoration and root dentin, which in turn results in their fracture.^35^^,^^36^ Cyclic loading tests that reproduce the forces repeatedly exerted by mastication and thermal cycling tests must be conducted to assess the effects of wet environments and temperature changes in the oral environment to further investigate the effectiveness of zirconia tubes in foundation restoration.^37^

Currently, due to the limitation of the fabrication of the zirconia tubes, the core build-up systems using zirconia tubes can only be applied to molars with large pulp chambers or teeth with widely enlarged root canals. It is necessary to conduct further research to determine the appropriate form of the zirconia tube, such as a tapered tube, so that this method can be applied to the teeth with narrow post space.

The use of a composite resin core with a zirconia tube for the simulated premolar with flared root canals reduced strain at the cervical surface area and provided higher fracture strength compared to using a composite resin core with a fiber post. And the zirconia tubes provided even higher fracture strength when used with a fiber post.

## Declaration of competing interest

The authors have no conflicts of interest directly relevant to the content of this article.
